# Public Awareness of Common Eye Diseases and the Role of Pharmacists in Raising This Awareness in Saudi Arabia: A Cross-Sectional Study

**DOI:** 10.3390/healthcare9060692

**Published:** 2021-06-08

**Authors:** Farhan Alshammari, Sameer Shaikh, Arshad Hussain, Ahmed Alafnan, Ibrahim Almuzaini, Bushra Alshammari

**Affiliations:** 1 Department of Pharmaceutics, College of Pharmacy, University of Hail, Hail 2440, Saudi Arabia; frh.alshammari@uoh.edu.sa; 2 Divisions of Oral Diagnosis and Oral Medicine, Department of OMFS and Diagnostic Sciences, College of Dentistry, University of Hail, Hail 2440, Saudi Arabia; 3 Department of Clinical Pharmacy, College of Pharmacy, University of Hail, Hail 2440, Saudi Arabia; ar.hussain@uoh.edu.sa (A.H.); brbrzz1998@gmail.com (I.A.); 4 Department of Pharmacology & Toxicology, College of Pharmacy, University of Hail, Hail 2440, Saudi Arabia; a.alafnan@uoh.edu.sa; 5 Department of Medical Surgical Nursing College of Nursing, University of Hail, Hail 2440, Saudi Arabia; Bu.Alshammari@uoh.edu.sa

**Keywords:** awareness of eye disorders, cataracts, diabetic retinopathy, dry eyes, general population, glaucoma, role of pharmacists, Saudi Arabia

## Abstract

Knowledge of common eye disorders and their prevention and treatment can play an essential role in reducing the visual impairment burden. A cross-sectional, questionnaire-based study was conducted from 15 November 2020 to 15 January 2021 to estimate the knowledge and awareness about common eye problems and their possible risk factors among the general population of the Hail Region, Saudi Arabia. The study also investigated the participants’ sources of information about eye diseases. Participants from various areas of the Hail Region were randomly selected. There were four parts in the questionnaire based upon the general awareness about the common eye diseases, participants’ knowledge and awareness of risk factors associated with eye disorders, awareness of treatment and prevention of various eye disorders, and the participants’ sources of information about ocular diseases. The questionnaire was distributed to the participants by direct contact with them at eye clinics, hospitals, malls, and markets. The number of participants in the survey was 400 people, 53.8% males and 46.3% females. Cataracts were known to 31% of the participants, 43% knew about glaucoma, 66% knew about dry eyes, and 44% knew about diabetic retinopathy. Overall, 46% of the participants knew about eye problems, and the male participants had better knowledge about eye problems than the female ones. The primary sources of information about the common eye diseases were pharmacists (43.3%), family physicians (40.3%), the Internet (37.3%), the community (37.3%), television and radio (9.3%), and books/ brochures (9.5%). In the Hail Region, the general public carries a moderate awareness of preventable vision-threatening ocular disorders. However, the awareness of cataracts and glaucoma was low. The gaps in awareness can be overcome by public health promotion. In offering information about eye conditions to the general public, pharmacists may play a crucial role.

## 1. Introduction

Good vision is an essential aspect of autonomous life. Globally, at least 1 billion people have near or distant vision impairments that could have been prevented or have not yet been treated [1]. The primary causes of visual impairment are uncorrected refractive errors followed by cataracts. Other visual impairment causes include glaucoma, diabetic retinopathy (DR), and age-related macular degeneration (AMD) [2]. Most individuals ignore early signs of eye diseases and fail to seek timely treatment to avoid permanent vision loss [3]. The unawareness of prevention or the delay in seeking medical care for ocular diseases, such as glaucoma or cataract, may cause blindness [4]. It is alarming that many older adults are misguided to believe that visual impairment and blindness are a normal part of the aging process [5]. The epidemiology of visual impairment can be linked to an increasing rise in non-communicable diseases, especially diabetes, and other lifestyle-related factors, including dietary changes, sedentary lifestyles, and smoking [2]. Increased community understanding and knowledge of common eye disorders and their treatment options are critical for promoting preventive ophthalmic care [6]. Cataract, the most prevalent visual impairment, is associated with aging, smoking, diabetes, ultraviolet radiation, and alcohol consumption [7]. Visual loss from glaucoma is permanent and is sometimes imperceptible at an early point. Globally, more than 60 million people are estimated to have glaucoma; yet, in developing countries, only around half of all glaucoma patients have been diagnosed [8]. Dry eyes, caused by inflammation and injury of the ocular surface, are a significant condition that can impair life quality. Prolonged tear evaporation and aqueous deficiency are the two underlying factors implicated in dry eyes. This specific ocular condition can lead to eye symptoms such as pain, redness, irritation, and blurred vision. The main risk factors associated with dry eyes’ pathogenesis are age, female gender, and cigarette smoking. Individuals with specific diseases such as Sjögren’s syndrome, rheumatoid arthritis, and diabetes mellitus remains at risk of dry eyes [9]. Diabetic retinopathy (DR) is a complication of diabetes and is among the leading causes of blindness [10]. The major goal of community pharmacy is to enhance the public’s health and quality of life, and there is evidence that community pharmacy-based services lead to better patient care and health outcomes [11]. Previous research [12,13] in Saudi Arabia found that many Saudi Arabian healthcare consumers visit their local community pharmacy for a variety of reasons, including disease-related counseling, the purchase of over-the-counter (OTC) medications or cosmetic items, and the acquisition of prescription medication for the treatment or prevention of the advancement of chronic illnesses. Moreover, Saudi Arabian healthcare consumers expect community pharmacists to play a substantial role in improving health outcomes [14]. Concerning the treatment and prevention of ocular diseases, community pharmacists can play an important role. In France, pharmacists actively participate in delivering eye illness counseling and patient treatment [15]. With pharmacists increasingly being the first port of call for many patients, community pharmacists can aid both in the detection of warning symptoms signaling potentially significant underlying eye diseases, as well as in the routine care of common eye diseases [14].

There is a scarcity of studies on eye diseases among the people of Northern Saudi Arabia, including the Hail Region. The current study aimed to estimate public awareness about common eye problems and the associated risk factors. Considering the emerging role of community pharmacists in preventing and managing ocular diseases, the contribution of pharmacists in raising awareness of the ocular diseases in the Hail Region is investigated through participants’ sources of information about common eye diseases.

## 2. Materials and Methods

A quantitative, questionnaire-based, and cross-sectional study was conducted over a two-month period from November 2020 to 15 January 2021. This study was reviewed and approved by the Research Ethics Committee (REC) of the University of Hail. The approval number was 16784/5/42. Participants from various areas of the Hail Region were randomly selected. Calculating the sample size using (Roasoft) program at a 5% margin of error and 95% confidence level, the minimum number of participants determined for this survey was 384. Faculty members from the College of Pharmacy, University of Hail, evaluated the questionnaire’s content validity. The questionnaire was piloted among a few randomly selected participants, and appropriate adjustments were made per the response. The first part of the questionnaire was about the study participants’ socio-demographic information and their general awareness about the common eye diseases. The second part was about participants’ knowledge and awareness of risk factors associated with eye disorders. The third part inquired about the awareness of treatment and prevention of various eye disorders. The last part of the questionnaire inquired about the participants’ sources of information about ocular diseases. Subjects above 18 years old from the Hail Region were eligible to participate in this analysis, regardless of sex, including pregnant women. Individuals with intellectual disabilities and those who cannot complete or answer the questionnaire were excluded. Written, informed consent was obtained from each patient after the aims and methodology of the study were explained to them. The questionnaire was distributed to the participants by direct contact with them at eye clinics, hospitals, malls, and markets. The participants were provided with sufficient time for onsite completion of the questionnaire. Microsoft Excel 2016 version (Microsoft, Redmond, WA, USA) was used to categorize the data. Categorical data were represented as numbers and percentages in brackets. Pearson’s chi-square test was used to evaluate and quantify the associations between the demographic variables and awareness of various eye disorders. SPSS version (IBM, Chicago, IL, USA) was used for analysis. A *p*-value of ≤0.001 was considered statistically significant.

## 3. Results

### 3.1. General Awareness about the Common Eye Diseases

**Figure 1:**
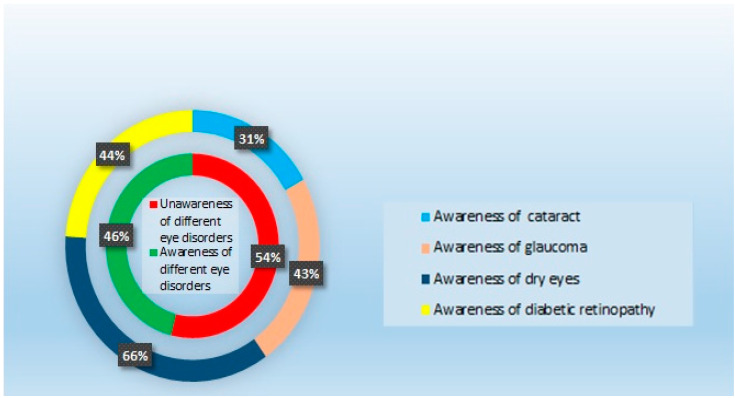
Participants’ awareness of eye disorders.

The number of participants in the survey was 400 people, 53.8% male and 46.3% female. The response rate of the participants was 100%. Thirty-one percent of participants knew of cataract, 43% had knowledge of glaucoma, 66% had knowledge of dry eyes, and 44% had knowledge of diabetic retinopathy. Overall, 46% of the participants were aware of the different eye problems, and an increased number of male participants (cataract (*n* = 87, *p* = 0.000); glaucoma (*n* = 108, *p* = 0.001); dry eyes (*n* = 151); diabetic retinopathy (*n* = 105)) were aware of the eye problems compared to the female participants (cataract (*n* = 36); glaucoma (*n* = 62); dry eyes (*n* = 111); diabetic retinopathy (*n* = 69)). Among the participants from different educational backgrounds, a greater number of undergraduates were aware of cataract (*n* = 86), glaucoma (*n* = 121), dry eyes (*n* = 176), and diabetic retinopathy (*n* = 122). Among the participants from different occupational backgrounds, students had a better awareness of cataract (*n* = 80), glaucoma (*n* = 100), dry eyes (*n* = 170), and diabetic retinopathy (*n* = 114) (Table 1 and Figure 1).

### 3.2. Participants’ Knowledge and Awareness of Risk Factors Associated with Eye Disorders

Compared with the awareness of risk factors associated with glaucoma and diabetic retinopathy, the awareness of risk factors associated with cataract was much lower (aging (*n* = 124, 31%); inherited genetic disorders (*n* = 81, 20.3%); systemic disease such as diabetes and hypertension (*n* = 105; 26.3%)). However, the long-term use of steroid medications as a risk factor for cataract was identified by 76.5% (*n* = 306) of participants (Table 2).

### 3.3. Awareness of Treatment and Prevention of Various Eye Disorders

The level of awareness of prevention (*n* = 87, 21.8%) and treatment (*n* = 121, 30.3%) of cataract was low in comparison to the awareness of prevention and treatment of dry eyes (prevention (*n* = 241; 60.3%; treatment (*n* = 256, 64%)) and diabetic retinopathy (prevention (*n* = 291, 72.8%); treatment (*n* = 290, 72.5%)). A considerable number of participants had the awareness of the prevention of glaucoma (*n* = 279, 69.8%), however 55% (*n* = 220) of the participants had the awareness of the reversibility of damage due to glaucoma. Participants’ awareness of treatment and prevention of various eye disorders are presented in Table 3.

### 3.4. Participants’ Sources of Information about Ocular Diseases

**Figure 2:**
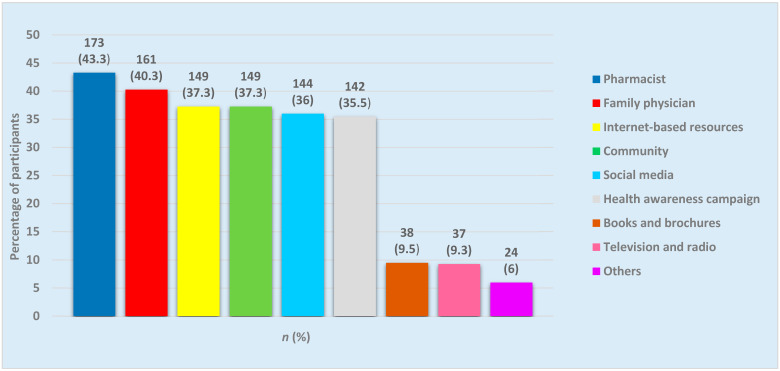
Participants’ sources of information about the eye diseases.

Participants’ sources of information about the eye diseases are shown in Figure 2. The most commonly reported sources were pharmacists (43.3%), family physicians (40.3%), internet-based resources (37.3%) and members of the community (37.3%).

## 4. Discussion

Visual impairment and blindness are significant global health challenges since they are related to morbidity, death, and lower quality of life, resulting in considerable economic loss and productivity [16].

In Saudi Arabia, the trend and causes of visual impairment in the past 40 years were influenced by significant socio-economic changes. The incidence of blindness reported in Saudi Arabia three decades ago was 10–20 times higher than that of Europe and the US. Despite the improvements in eye healthcare services, low vision and blindness remain a formidable health challenge in Saudi Arabia [17,18]. The knowledge of common eye disorders and an understanding of these disorders’ prevention and treatment is essential in motivating patients to seek timely eye care, which eventually helps to reduce the burden of vision impairment. Studies from northern Saudi Arabia on public health awareness of eye-related diseases and eye health education are scarce. To fill the research gap, the current study aimed to determine the awareness about common eye problems and their possible risk factors among the general population in the Hail Region, which is located in the northern part of Saudi Arabia. To the best of our knowledge, the current study appears to be the only study from Northern Saudi Arabia to investigate the general awareness of the risk factors, prevention, and treatment of the common eye disorders. Overall, 46% of the respondents had a general awareness of the various eye issues including, dry eyes, diabetic retinopathy, glaucoma, and cataract. In comparison to the female participants, the male participants had a better level of awareness of eye diseases. This finding was in contrast to the study findings conducted in Riyadh, Saudi Arabia, in which female adults on average had better knowledge regarding eye diseases and eye care [19]. Social restrictions in the Hail Region can be attributed as the factor for limited access of females to various information sources about eye disorders. However, a study in Nepal showed better knowledge among males compared to females [20]. A better understanding of eye disorders among males in the Hail Region can be attributed to their willingness to seek information about such disorders. Moreover, in comparison to females, males in the Hail Region have easier access to various information sources about eye disorders. In the current study, among participants from different occupational backgrounds, students had a better awareness of cataracts, glaucoma, dry eyes, and diabetic neuropathy. Similarly, in a study to assess the public awareness of common ocular diseases such as glaucoma, cataract, diabetic retinopathy, and dry eye in Jordan, the level of education of participants was significantly associated with a better awareness of these eye disorders [4]. Interestingly, fewer participants (31%) in the current study were aware of the cataracts. This finding was in contrast to the results of studies from Bangladesh and Canada. In Bangladesh, most participants (90%) had heard of cataracts, while in Canada, 69.2% reported familiarity with cataracts as a cause of vision loss [2,21].

Moreover, the current study participants showed a lack of awareness about the risk factors (aging, smoking, and systemic diseases) associated with cataracts. On a similar note, the awareness of the possibility of prevention (21.8%) and treatment (30.3%) for cataract was found to be low in comparison to glaucoma (prevention (69.3%); reversibility of glaucoma damage (55%)), dry eyes (prevention (60.3%); treatment (64%)) and diabetic retinopathy (prevention (72.8%); treatment (72.5%)). In Makkah, Saudi Arabia, most of the respondents (72.4%) did not know that cataract is an increase in the lens’s opacity, and 78% did not know that cataract can lead to blindness [22]. Like other arid areas of Saudi Arabia, the Hail Region has a hot and dry climate. A hot and dry atmosphere has been believed to aggravate various dry eyes’ symptoms such as gritty, sandy, and foreign body sensations in the eyes. This factor can have relevance in better understanding of dry eyes, their prevention, and treatment among the Hail Region respondents [23]. The current study was significant because, through it, community pharmacists’ role in raising the awareness of ocular diseases came to the fore. In the current study, pharmacists were the leading source of information about eye diseases, followed by family physicians and internet-based resources. This finding gives credence to the proposal to broaden the pharmacist’s role beyond the conventional product-oriented roles of dispensing and distributing medications and health supplies. Pharmacists were active players in offering guidance on eye diseases and in caring for patients in France [15]. The same model can be followed in Gulf Cooperation Council countries, including Saudi Arabia, by involving the community pharmacists in counseling the patients and the general population about various aspects of ocular diseases, including prevention and treatment. The counseling pharmacist should have sufficient knowledge and be an efficient communicator, using verbal and non-verbal communication skills to help the masses to better understand ocular diseases. Continuing education from pharmaceutical companies and postgraduate education on ocular diseases and treatment can establish the community pharmacists as active players in providing advice on ocular diseases and taking care of patients.

### 4.1. Recommendations

Community pharmacies are highly accessible across Saudi Arabia, and without appointments or referrals, it is easy for health care customers to approach the community pharmacist. In addition, Saudi health care customers believe that community pharmacists can significantly improve health outcomes. Pharmacists’ participation in increasing the awareness of public health at the community level, informing the public about the prevention and control of eye diseases, and improving the quality of medicinal products may also reduce visual impairment. To achieve this aim, the profession of community pharmacy in Saudi Arabia requires many improvements to enhance its public profile. This includes the pharmacy’s separation from the drug store and redesigning the pharmacy to include a private room for consultation. These improvements can only be implemented if they are accompanied by changes to the current Saudi Arabian pharmacy rules, the inclusion of pharmacists in health care system planning, and the recognition of pharmacies as a clinical career [11,24]. Moreover, in order to improve the access women have to information about ocular diseases and its prevention, community pharmacists can work collaboratively in a gender-sensitive fashion within communities (for example, collaboration with women’s groups, traditional healers, and local service groups) [25].

### 4.2. Limitations and Strengths of the Study

A brief duration of two months for data collection was a limiting factor in the current study. A longer duration of data collection would have helped generate a more comprehensive determination of general awareness related to eye disorders in the Hail Region. Together with this limitation, there were a few strengths of this study. The current study can be termed as the first study to explore the comprehension of preventable sight-threatening eye disorders among the general population from Northern Saudi Arabia. Moreover, it is the only study that elaborated on community pharmacists’ role in raising awareness among the general population of preventable sight-threatening ocular diseases.

## 5. Conclusions

In the Hail Region of Saudi Arabia, there is an overall moderate comprehension of common eye disorders among the general population. Of most interest was the low comprehension of preventable sight-threatening eye disorders, particularly cataracts and glaucoma. Our results indicate that the general population should be targeted by health awareness and public health initiatives for eye care. In Saudi Arabia, educational programs to increase public knowledge of ocular diseases can improve the effectiveness of health promotion, thereby preventing unnecessary blindness. As an integral member of the healthcare team, community pharmacists can play a crucial role in raising awareness of eye disease prevention and treatment.

## Figures and Tables

**Table 1:** Association between demographic data and level of awareness of various eye disorders.

Variables	*n* (%)	Familiarity with Various Eye Disorders											
		Cataract			Glaucoma			Dry Eye			Diabetic Retinopathy		
		Yes *n*	No *n*	*p*	Yes *n*	No *n*	*p*	Yes *n*	No *n*	*p*	Yes *n*	No *n*	*p*
**Gender**													
Male	215 (53.8)	87	128	≤0.001	108	107	≤0.001	151	64	0.035	105	110	0.026
Female	185 (46.3)	36	149		62	123		111	74		69	116	
**Marital status**													
Single	263 (65.8)	74	189	0.274	97	166	0.013	170	93	0.647	102	161	0.039
Married	127 (31.8)	46	81		67	60		87	40		65	62	
Divorced	6 (1.5)	1	5		3	3		3	3		4	2	
Widowed	4 (1.0)	2	2		3	1		2	2		3	1	
**Educational level**													
Primary school	1 (0.3)	0	1	0.007	0	1	0.087	0	1	0.394	0	1	0.431
Secondary school	10 (2.5)	1	9		2	8		7	3		4	6	
Higher secondary school	88 (22.0)	19	69		31	57		63	25		33	55	
Undergraduate	274 (68.5)	88	186		121	153		176	98		122	152	
Postgraduate	27 (6.8)	15	12		16	11		16	11		15	12	
**Occupation**													
Student	263 (66.8)	80	183	0.814	100	163	0.025	170	93	0.255	114	149	0.482
Employed	102 (25.5)	34	68		52	50		69	33		48	54	
Unemployed	29 (7.3)	7	22		13	16		17	12		9	20	
Self-employed	6 (1.5)	2	4		5	1		6	0		3	3	

**Table 2:** Awareness of different risk factors associated with various eye disorders.

Risk Factors Associated with Various Eye Disorders	Yes *n* (%)	No *n* (%)
**CATARACT**		
Aging	124 (31.0)	276 (69.0)
Smoking	71 (17.8)	329 (82.3)
Inherited genetic disorders	81 (20.3)	319 (79.8)
Long-term use of steroid medications	306 (76.5)	94 (23.5)
Systemic diseases such as diabetes and hypertension	105 (26.3)	295 (73.8)
**GLAUCOMA**		
Age above 60 years	248 (62.0)	152 (38.0)
Family history of glaucoma	300 (75.0)	100 (25.0)
High internal eye pressure (intraocular pressure)	120 (70.0)	280 (30.0)
Systemic diseases such as diabetes and hypertension	269 (67.3)	131 (32.8)
**DRY EYES**		
Old age	277 (69.2)	123 (30.8)
Exposure to wind, smoke, or dry air	245 (61.2)	155 (38.8)
Female gender	316 (79.0)	84 (21.0)
Medications including antihistamines and antidepressants	222 (55.5)	178 (44.5)
Certain medical conditions such as Sjogren’s syndrome and rheumatoid arthritis	276 (69.0)	124 (31.0)
**DIABETIC RETINOPATHY**		
Duration of diabetes	270 (67.5)	130 (32.5)
Poor control of your blood sugar level	240 (60.0)	160 (40.0)
Smoking	60 (15.0)	340 (85.0)
High blood pressure/high cholesterol	240 (60.0)	160 (40.0)

**Table 3:** Awareness of the treatment and prevention of various eye disorders.

Question	Yes *n* (%)	No *n* (%)
**CATARACT**		
Is cataract preventable?	87 (21.8)	313 (78.3)
Is cataract treatable?	121 (30.3)	279 (69.8)
**GLAUCOMA**		
Is glaucoma preventable?	279 (69.8)	121 (30.3)
Is the damage due to glaucoma reversible?	220 (55.0)	180 (45.0)
**DRY EYES**		
Are dry eyes preventable?	241 (60.3)	159 (39.8)
Are dry eyes treatable?	256 (64.0)	144 (36.0)
**DIABETIC RETINOPATHY**		
Is diabetic retinopathy preventable?	291 (72.8)	109 (27.3)
Is diabetic retinopathy treatable?	290 (72.5)	110 (27.5)

## Data Availability

The data presented in this study are available on request from the corresponding author after university approval. The data are not publicly available due to privacy or ethical restrictions.
